# Body mass index and height over three generations: evidence from the Lifeways cross-generational cohort study

**DOI:** 10.1186/1471-2458-12-81

**Published:** 2012-01-25

**Authors:** Celine M Murrin, Gabrielle E Kelly, Richard E Tremblay, Cecily C Kelleher

**Affiliations:** 1 School of Public Health, Physiotherapy and Population Science, University College Dublin, Woodview House, Belfield, Dublin 4, Ireland; 2 School of Mathematical Sciences, University College Dublin, Library Building, Belfield, Dublin 4, Ireland

## Abstract

**Background:**

Obesity and its measure of body mass index are strongly determined by parental body size. Debate continues as to whether both parents contribute equally to offspring body mass which is key to understanding the aetiology of the disease. The aim of this study was to use cohort data from three generations of one family to examine the relative maternal and paternal associations with offspring body mass index and how these associations compare with family height to demonstrate evidence of genetic or environmental cross-generational transmission.

**Methods:**

669 of 1082 families were followed up in 2007/8 as part of the Lifeways study, a prospective observational cross-generation linkage cohort. Height and weight were measured in 529 Irish children aged 5 to 7 years and were self-reported by parents and grandparents. All adults provided information on self-rated health, education status, and indicators of income, diet and physical activity. Associations between the weight, height, and body mass index of family members were examined with mixed models and heritability estimates computed using linear regression analysis.

**Results:**

Self-rated health was associated with lower BMI for all family members, as was age for children. When these effects were accounted for evidence of familial associations of BMI from one generation to the next was more apparent in the maternal line. Heritability estimates were higher (h^2 ^= 0.40) for mother-offspring pairs compared to father-offspring pairs (h^2 ^= 0.22). In the previous generation, estimates were higher between mothers-parents (h^2 ^= 0.54-0.60) but not between fathers-parents (h^2 ^= -0.04-0.17). Correlations between mother and offspring across two generations remained significant when modelled with fixed variables of socioeconomic status, health, and lifestyle. A similar analysis of height showed strong familial associations from maternal and paternal lines across each generation.

**Conclusions:**

This is the first family cohort study to report an enduring association between mother and offspring BMI over three generations. The evidence of BMI transmission over three generations through the maternal line in an observational study corroborates the findings of animal studies. A more detailed analysis of geno and phenotypic data over three generations is warranted to understand the nature of this maternal-offspring relationship.

## Background

Parental body mass index (BMI) is the most powerful determinant of offspring's BMI [1-3]. Family and twin studies have demonstrated a clear heritability pattern, with estimates ranging from 50% to 90% [4]. Inheritance of BMI is thought to arise from a combination of both inherited genes and shared environment where both parents contribute equally to body composition [5,6]. However, numerous two-generational studies have reported stronger associations for mothers than for fathers [4,7-10], which suggests that mothers bear a unique influence on offspring body composition, possibly through intrauterine mechanisms. Evidence of an enduring multigenerational pattern of BMI may provide a clearer demonstration of the relative influence of maternal and paternal contributions to offspring BMI.

Whilst most observational studies have demonstrated inheritance of BMI through two generations, as yet, no human studies have demonstrated the relationship across three generations. Familial patterns of birth weight across multiple generations have been reported [11-13] in addition to trans-generational responses to food availability and longevity, [14] but prospective studies of three generations are scarce. While several large cohort studies have information on three generations, the grandparental-parental-child relationship has either not yet been reported or does not describe the maternal and paternal lines distinctly [15,16]. To allow for a greater understanding of the contribution of parental body composition to offspring BMI, a simple approach is to report weight, height and BMI for all family members since, relative to weight and BMI, height demonstrates much clearer evidence of genetic variation inherited from both parents [17,18]. No studies have examined simultaneously the associations of weight, height and BMI across three generations of both maternal and paternal lines.

The primary objective of this study was to fill this gap with data from the Lifeways longitudinal study. The associations of weight, height, and body mass index over three generations were analysed. The second objective was to compare the associations of weight and BMI within families with that of height to identify evidence of genetic or environmental transmission while controlling for other factors including, diet, physical activity, and socioeconomic status.

## Methods

### Participants

This prospective longitudinal study was established in an 18 month period of 2001-2003 when a random sample of 1124 expectant Irish-born mothers were recruited while attending their first ante-natal visit [19,20]. The cohort is comprised of the index mother and child, and where agreeable, the father, and at least one grandparent randomly selected on a rotated basis from a full list of all available grandparents, maternal grandmother (MGM), maternal grandfather (MGF), paternal grandmother (PGM), and paternal grandfather (PGF) [21]. At recruitment mothers were asked to complete a questionnaire with sections relating to health, diet, lifestyle factors, demographic, occupation, social, and living characteristics [19]. Mothers were asked to report their height and weight before they became pregnant and BMI (weight (kg)/height (m^2^)) was subsequently calculated. Fathers and grandparents were asked to complete a shorter version of the same questionnaire where they also reported their height and weight [19].

A follow-up study of the families was conducted in a 9 month period of 2007-2008 when the children were aged 5 years on average. Of the 1124 mothers who consented to the study at baseline, 1082 families with 1094 live infants were invited to participate in the follow-up. This number included 12 children, born with varying degrees of birth abnormalities, who were contacted separately. Of the 1082 families, 669 of mothers (62%) responded to the follow-up. Mothers who remained in the study did not report any significant difference in BMI at baseline from those who were no longer in the study [22]. Mothers completed a follow-up questionnaire from baseline which was expanded to provide information on their child's health, physical activity, and diet [22].

Children's weight and height were measured at home to the nearest 0.1 kg and 1 cm by a team of researchers trained using standardized protocols [23,24]. Height was measured using a Leicester portable stadiometer and weight a Tanita Digital Weighing Scale Model HD305 (both sourced and calibrated by Chasmors Ltd., Camden High Street, London). Height and weight measures were used to calculate BMI. Age and sex specific BMI standards and the International Obesity Taskforce cut-offs that correspond to BMI of 25 or 30 kg/m^2 ^at age 18 years were used to identify overweight and obese children [25,26]. In adults overweight was defined as a BMI between 25 and 29.9 kg/m^2 ^and obesity was defined as a BMI greater than 30 kg/m^2^. Weight, height and BMI were the outcomes of interest. The sample used in this analysis was the cohort of children at follow-up who had complete questionnaire information and measurement information (n = 585). Twins (n = 10) and children whose mothers were pregnant or up to 6 weeks postpartum at follow-up were excluded from the analysis resulting in a final sample of n = 529 children.

### Risk factors

The risk factors were selected for the main analysis on the basis that the same variable, derived from the baseline questionnaire, was available for each family member. Indicators of socioeconomic status used were level of education (None/primary, Second level, Third level) and medical card status (No medical card versus medical card holder where eligibility to free medical care is on an income basis and was previously used as a good indicator of social disadvantage in an Irish sample) [27]. These two variables were used in duplicate for both mother and child as, at this age, the child may not have started formal education and would be dependent on the mother's general medical services. Dietary intakes were assessed using a standardized Food Frequency Questionnaire (FFQ) which contains 149 food items and responders are asked to report their average use of each food item over the previous year or, in the case of mothers, since they became pregnant [19]. Two proxy measures for general healthy diet were created from the FFQ on the basis of adherence to dietary guidelines. At present, the recommendation for fruit and vegetable intake is 5 or more portions per day and intake of oily fish at least once a week. The proxy variable for fruit and vegetables was created using a sum of average daily intakes which was then dichotomised into those who did or did not meet the recommendations (fruitveg). Average daily intakes of oily fish were also computed and responses categorised into those who were regular consumers (at least once as week), occasional consumers and those who never consumed oily fish. Other variables included in the analysis were self reported physical activity level which was created from responders engagement in light, moderate or heavy physical exercise. Each individual was then graded on their frequency and level of physical activity (based on a score of 0 to 18) and then categorised into one of four groups (none, low, medium, and high levels of physical activity). Finally self-rated health (SRH) was used as an indicator of overall health status and the original five response options were collapsed to those who reported excellent or very good health, good or fair health, and poor health.

### Statistical analyses

Independent t-tests and χ^2 ^tests were used to investigate for the presence of patterns in the missing values which might influence quantitative outcomes.

We carried out descriptive analyses of weight, height, and BMI and *t*-test for gender differences among children in each age group. Correlation coefficients between family members were calculated for weight, height and BMI. Univariate linear regression analysis was conducted for child weight, height, BMI, and family data. Parent-offspring linear regression analyses were further employed to estimate to what extent the phenotypic variance (V_P_) in BMI was attributable to genetic (V_G_) versus environmental factors (V_E_). The proportion of the phenotypic variance attributable to additive genetic variance (V_A_) was estimated using narrow-sense heritability (h^2^) where h^2 ^= V_A_/V_P_. Heritability estimates were calculated by conducting a linear regression with each child and parent/grandparent pair. The slope of the regression line or the regression co-efficient is used to estimate the association between the two parents and their offspring, Therefore twice the regression co-efficient was used to approximate offspring-parent heritability of height, weight and BMI [28].

A mixed model analysis was conducted for, at a minimum, both child and mother (n = 454). The maximum number of individuals in a family was seven: child, mother, father, maternal grandmother (MGM), maternal grandfather (MGF), paternal grandmother (PGM), paternal grandfather (PGF), or groups numbered 1 to 7. Each family had a unique identifier (family id). Each family member provided at least one record for the dataset and the resulting dataset had n = 3703 records. Thus, it was possible to include data on incomplete families in the models. BMI was the outcome measure in mixed model. Family id was fitted as a random effect and BMI measures on individuals in the same family were assumed correlated i.e. BMI measures between family members of the different groups were assumed correlated. Different correlation structures were considered: unstructured (un), compound symmetry (cs), heterogenous compound symmetry (csh), un(1), which fits different variances in each group but zero correlation between family members, and the best one chosen using Akaike's Information Criterion (AIC) or a likelihood ratio test in the case of nested structures.

The fixed effect variables considered for modelling were: gender, group, age, self-rated health (SRH), education, medical card holder (GMS), fruit & vegetables and fish consumption, physical activity level, and interactions of these variables. Stepwise procedures were used to find the fixed effects that provided the best model. *P*-values < 0.05 are regarded as significant.

The estimated correlations between family members from the models represent the correlations when fixed effects common to all individuals are removed. The Bonferroni correction was used to adjust *p*-values arising from multiple correlations between groups. Confidence intervals based on Fisher's z transform, rather than standard errors are reported for correlations as correlation estimates typically are not symmetrically distributed.

A similar model was fitted with height as the outcome variable. In addition, each individual was classified as obese/not obese and a model fitted to this binary outcome variable using the same fixed and random effects as in the models described above.

Models were fitted using the SAS version 9.1.3 statistical procedures Mixed and Glimmix.

Ethical approval for the Lifeways Cohort and follow-up was granted by ethics committees in the National University of Ireland, Galway; The Coombe Women's Hospital, Dublin; University College Hospital, Galway; The Irish College of General Practitioners.

## Results

No significant patterns were found among missing data so it is assumed the data is missing completely at random.

Descriptions of weight, height and BMI of each family member are reported in Table 1. With the exception of mothers' BMI, the mean BMI for other adult members falls within the overweight category (25 to 29.9 kg/m^2^). The majority of the children were aged 5 years (Table 2) and significant differences between boys and girls were only found in the weight of the 6 year olds where the mean weight of girls was higher than that of boys (Table 2). More than a quarter of children and mothers were either overweight or obese which increased to approximately 60 to 70% for other family members (Table 3).

**Table 1:** Mean age, height weight and BMI of family members in Lifeways

			Age (years)		Height (cm)		Weight (kg)		Body mass index (kg/m^2^)	
**Cohort member**	**n**	**Group**	**Mean**	**SD**	**Mean**	**SD**	**Mean**	**SD**	**Mean**	**SD**

Boys	260	1	5.37	0.32	112.05	4.85	20.92	2.82	16.63	1.7

Girls	269	1	5.36	0.29	112.04	4.98	20.91	3.12	16.61	1.83

Mother	454	2	30.85	5.70	163.79	6.35	63.46	10.81	23.68	3.81

Father	191	3	34.42	5.65	178.51	7.05	84.04	13.12	26.34	3.78

MGM	147	4	60.50	8.54	161.13	6.90	68.48	13.16	26.36	4.27

MGF	98	5	63.33	9.09	172.96	7.04	80.54	12.66	26.99	4.00

PGM	84	6	62.10	8.74	160.75	6.54	68.32	11.76	26.64	4.37

PGF	53	7	62.85	10.10	172.10	8.68	82.20	15.04	28.12	5.06

MGM: Maternal grandmother, MGF: Maternal grandfather, PGM: Paternal grandmother, PGF: Paternal grandfather

**Table 2:** Mean height, weight and BMI by age and gender of children in Lifeways

				Gender			
	**Age (Years)**		**Boys**			**Girls**	

		**n**	**Mean**	**SD**	**n**	**Mean**	**SD**

Height (cm)	< 5	33	109.88	5.02	28	110.40	5.11

	5 to 6	214	112.11	4.70	235	112.02	4.80

	> 6	13	116.52	3.69	6	120.22	3.81

Weight (kg)	< 5	33	20.25	2.79	28	20.54	3.31

	5 to 6	214	20.96	2.84	235	20.84	3.03

	> 6	13	22.03	2.46	6	25.42	2.90*

Body mass index (kg/m^2^)	< 5	33	16.70	1.31	28	16.83	2.34

	5 to 6	214	16.65	1.77	235	16.56	1.77

	> 6	13	16.20	1.44	6	17.53	1.05

*T-test significant at the 0.05 level

**Table 3:** Percentage overweight and obese family members in Lifeways

			Overweight		Obese	
**Cohort member**	**n**	**Group**	**n**	**%**	**n**	**%**

Boys	260	1	49	18.8	17	6.5

Girls	269	1	61	22.7	21	7.8

Mother	454	2	92	20.3	36	7.9

Father	191	3	96	50.3	29	15.2

MGM	147	4	63	42.9	24	16.3

MGF	98	5	48	49.5	17	17.5

PGM	84	6	39	46.4	17	20.2

PGF	53	7	21	39.6	17	32.1

MGM: Maternal grandmother, MGF: Maternal grandfather; PGM: Paternal grandmother; PGF: Paternal grandfather

Univariate analysis of family BMI and weight over three generations showed significant relationships in the maternal line only (Table 4 and 5) whereas height was found to be significantly correlated across all family members in both maternal and paternal lines (Table 6). A similar familial pattern was demonstrated for heritability estimates of these phenotypes with significant associations found only in the maternal line for weight and BMI. Effect sizes were greater for height indicating that a significant proportion of the variation in height is attributable to heritable factors (Table 7).

**Table 4:** Univariate correlation matrix of family BMI

					Body Mass Index (kg/m^2^)						
		**Child**	**Mother**		**Father**	**MGM**		**MGF**		**PGM**	**PGF**

Child	r	1.000	0.200	**	0.111	0.201	*	-0.122		-0.074	0.119
	
	P		0.000		0.126	0.015		0.232		0.506	0.396
	
	n		454		191	147		98		84	53

Mother	r		1.000		0.075	0.269	**	0.302	**	0.061	-0.002
	
	P				0.330	0.002		0.005		0.609	0.884
	
	n				171	128		83		73	45

Father	r				1.000	-0.188		0.075		0.085	-0.059
	
	P					0.125		0.599		0.591	0.779
	
	n					68		51		42	25

MGM	r					1.000		-0.002		-0.039	-0.135
	
	P							0.990		0.847	0.594
	
	n							65		27	18

MGF	r							1.000		-0.019	0.214
	
	P									0.933	0.379
	
	n									23	19

PGM	r									1.000	0.099
	
	P										0.565
	
	n										36

PGF	r										1.000
	
	P										
	
	n										

**Correlation is significant at the 0.01 level (2-tailed), *Correlation is significant at the 0.05 level (2-tailed)

MGM: Maternal grandmother; MGF: Maternal grandfather; PGM: Paternal grandmother; PGF: Paternal grandfather

**Table 5:** Univariate Correlation matrix of family weight

						Weight (kg)						
		**Child**	**Mother**		**Father**		**MGM**		**MGF**		**PGM**	**PGF**

Child	r	1.000	0.216	**	0.298	**	0.192	*	0.049		0.137	0.215
	
	P		0.000		0.000		0.015		0.627		0.194	0.111
	
	n		467		201		160		100		92	56

Mother	r		1.000		0.074		0.286	**	0.342	**	0.078	0.047
	
	P				0.320		0.000		0.001		0.484	0.739
	
	n				181		145		90		83	52

Father	r				1.000		-0.219		0.057		0.136	0.304
	
	P						0.054		0.683		0.368	0.123
	
	n						78		54		46	27

MGM	r						1.000		0.203		-0.105	-0.042
	
	P								0.092		0.569	0.854
	
	n								70		32	22

MGF	r								1.000		0.071	0.241
	
	P										0.741	0.320
	
	n										24	19

PGM	r										1.000	0.241
	
	P											0.151
	
	n											37

PGF	r											1.000
	
	P											
	
	n											

**Correlation is significant at the 0.01 level (2-tailed), *Correlation is significant at the 0.05 level (2-tailed)

MGM: Maternal grandmother; MGF: Maternal grandfather; PGM: Paternal grandmother; PGF: Paternal grandfather

**Table 6:** Univariate correlation matrix of family height

							Height (cm)							
		**Child**	**Mother**		**Father**		**MGM**		**MGF**		**PGM**		**PGF**	

Child	r	1.000	0.329	**	0.413	**	0.166	*	0.196	*	0.248	*	0.316	*
	
	P		0.000		0.000		0.031		0.036		0.015		0.012	
	
	n	529	501		208		169		114		96		62	

Mother	r		1.000		0.208	**	0.357	**	0.426	**	0.097		0.127	
	
	P				0.003		0.000		0.000		0.359		0.350	
	
	n				201		160		108		91		56	

Father	r				1.000		0.156		0.219		0.357	**	0.279	
	
	P						0.167		0.096		0.007		0.136	
	
	n						80		59		55		30	

MGM	r						1.000		0.376	**	-0.082		-0.452	
	
	P								0.001		0.633		0.020	
	
	n								80		36		26	

MGF	r								1.000		0.088		0.111	
	
	P										0.662		0.615	
	
	n										27		23	

PGM	r										1.000		0.422	*
	
	P												0.005	
	
	n												42	

PGF	r												1.000	
	
	P													
	
	n												62	

**Correlation is significant at the 0.01 level (2-tailed), *Correlation is significant at the 0.05 level (2-tailed)

MGM: Maternal grandmother; MGF: Maternal grandfather; PGM: Paternal grandmother; PGF: Paternal grandfather

**Table 7:** Univariate linear regression and estimate of heritability of children's weight, height, and BMI respectively with family weight, height and BMI

	Weight					Height					BMI				
	
	Estimate	*p *value	R Square	Heritability		Estimate	*p *value	R Square	Heritability		Estimate	*p *value	R Square	Heritability	
Child-Mother	0.22	0.00	0.05	0.43	**	0.33	0.00	0.11	0.66	**	0.20	0.00	0.04	0.40	**

Child-Father	0.30	0.00	0.09	0.60	**	0.41	0.00	0.17	0.82	**	0.11	0.13	0.01	0.22	

Child-MGM	0.19	0.01	0.04	0.38	*	0.16	0.03	0.03	0.32	*	0.20	0.01	0.04	0.40	*

Child-MGF	0.05	0.63	0.00	0.10		0.20	0.04	0.04	0.40	*	-0.12	0.23	0.01	-0.24	

Child-PGM	0.14	0.19	0.02	0.27		0.25	0.01	0.06	0.50	*	-0.07	0.51	0.00	-0.14	

Child-PGF	0.22	0.11	0.05	0.43		0.32	0.01	0.10	0.64	*	0.12	0.40	0.01	0.24	

Mother-MGM	0.29	0.00	0.08	0.57	**	0.36	0.00	0.13	0.72	**	0.27	0.00	0.07	0.54	**

Mother-MGF	0.34	0.00	0.12	0.68	**	0.43	0.00	0.18	0.86	**	0.30	0.01	0.09	0.60	*

Father-PGM	0.14	0.37	0.02	0.27		0.36	0.01	0.13	0.72	*	0.09	0.59	0.01	0.17	

Father-PGF	0.30	0.12	0.09	0.61		0.28	0.14	0.08	0.26		-0.02	0.88	0.00	-0.04	

**Significant at the 0.01 level *Significant at the 0.05 level

### Mixed models of familial BMI

A model was fitted to BMI with the explanatory variables listed previously. The unstructured covariance fitted best among the covariance types and when compared to a model with no correlation terms the result was significant (*p *= 0.0177). The final model (Table 8a) included a group effect (*p *= 0.0005), age by group effect (*p *= 0.0256) and Self-rated health (SRH) by age (*p *= -0.0342). Reported Excellent/Very good SRH lowered BMI by 1.26 compared to Good/Fair SRH and Poor SRH and in addition Excellent/Very good SRH lowered BMI by 0.08 for every year of age. There was no effect of gender on the BMI of the child. There was an effect of age for children only, with each year of age lowering BMI by 0.57 (*p *= 0.0294). The differences in BMI between groups differed according to whether fruit and vegetable recommendations were met or unmet. The BMI of child, mother and MGM were all significantly positively correlated. The model fitting procedure was repeated for log (BMI) but there was no improvement in the fit by AIC.

**Table 8:** Estimates and standard errors of covariates in the best fitting mixed models fitted to BMI

Effect	Group	Estimate	Standard error	*p*-value		Correlation	95% CI	*P*-value
**Model a) Fitted to all family members ‡**								
Child	1	19.56	1.41		Child and Mother	0.17	(0.06, 0.26)	0.0015

Mother	2	22.08	1.11		Child and MGM	0.25	(0.08, 0.42)	0.0054

Father	3	35.07	5.47		Child and MGF	-0.23	(-0.43,-0.01)	0.0409

MGM	4	26.23	2.73		Mother and MGM	0.28	(0.10, 0.44)	0.0021

MGF	5	30.44	3.04		Mother and MGF	0.25	(0.01, 0.46)	0.0408

PGM	6	30.86	3.99					

PGF	7	24.65	5.13					

SRH 0 vs 1 & 2		-1.26	1.77					

**Effect of age within each group**								

Age*Child		-0.57	0.26	0.0294				

Age*Mother		0.04	0.03	0.16				

Age*Father		-0.23	0.13	0.0842				

Age*MGM		0.004	0.04	0.9126				

Age*MGF		-0.05	0.05	0.2572				

Age*PGM		-0.08	0.06	0.2274				

Age*PGF		0.11	0.08	0.1789				

Age*SRH (SRH = 0)		-0.08	0.04	0.0342				

Group 7 (PGF): Fruitveg 0 vs 1		-4.93	-1.67	0.0032				

**Model b) Maternal line**§**‡**								

SRH 0 vs 1&2		-3.33	1.23	0.0071	Child and Mother	0.18	(0.08, 0.28)	0.003

					Child and MGM	0.23	(0.06, 0.39)	0.0078

					Child and MGF	-0.23	(-0.43,-0.02)	0.028

					Mother and MGM	0.29	(0.12, 0.44)	0.0009

					Mother and MGF	0.25	(-0.02, 0.41)	0.0733

Model c) Paternal line†								

SRH 0 vs 1&2		-3.2	1.48	0.0308				

Group 7 (PGF): Fruitveg 0 vs 1		-4.92	1.66	0.0036				

**Effect of age within each group**								

Age*Child		-0.55	0.26	0.0352				

Age*Father		-0.247	0.135	0.07				

Age*PGM		-0.095	0.064	0.1407				

Age*PGF		0.072	0.814	0.3742				

Also shown are the significant ( *p *< 0.05) correlation estimates between family members.

MGM: Maternal grandmother, MGF: Maternal grandfather, PGM: Paternal grandmother, PGF: Paternal grandfather

§ There was a negative effect of age on BMI but only borderline significance *p *= 0.075

† Model with vs without correlation terms *p *< 0.05; ‡Model with vs without correlation terms *p *< 0.0001

The variables medical card holder (GMS), fish consumption, and physical activity level were not significant in this model or any of the later models.

#### BMI Maternal line

A similar model was fitted separately to the maternal line (only including child, mother, MGM and MGF (Table 8b). The unstructured correlation fitted best and when compared to a model with no correlation terms the result was significant (*p *< 0.0001). The correlations found to be significant in the family model remained significant in the maternal line model with similar estimates. The group term was significant (*p *< 0.0001). Excellent/Very good SRH had lower BMI than Good/Fair SRH and Poor SRH by 3.33(*p *= 0.0071). For children there was a negative effect of age with BMI lowered by 0.45 for each year of age but this effect had only borderline significance (*p *= 0.0750). The correlation estimates between child, mother and MGM ranged from 0.18 to 0.29 (adjusted *p*-value < 0.05 for all estimates).

#### BMI Paternal line

A model was fitted separately to the paternal line also (only including child, father, PGM and PGF) (Table 8c). There was no correlation between family members as indicated by the models with unstructured covariance or csh. The model that fitted best had covariance structure un (1). For the fixed effects, the group term was significant (*p *< 0.0001) as to be expected. SRH was significant *p *= 0.0308; Excellent/Very good SRH had lower BMI than Good/Fair and Poor SRH by 3.20 (*p *= 0.0308). The interaction of group with fruit/veg was significant. The differences in BMI between groups differed according to whether fruit/veg was 0 or 1. Paternal grandfathers who did not meet fruit and vegetable recommendations BMI lowered by 4.92 compared to those meeting the recommendations (*p *= 0.0036).

In Table 8*p*-values for correlation coefficients need to be adjusted for multiple comparisons. If the conservative Bonferroni correction is used, then each *p*-value in model a) needs to be multiplied by 21 and in models b) and c) by 6.

### Mixed models of familial height

#### Height maternal line

Using the same procedures as for BMI, models were fitted to heights of family members and the results are shown in Table 9. In the analysis of height for the maternal line, the unstructured correlation matrix fitted best and overall the correlations between family members were significant (*p *< 0.0001). Heights of child and mother, child and MGM, child and MGF, and mother and MGF were all positively correlated. Applying the Bonferroni correction the correlation between child and mother, mother and MGF remain while child and MGF becomes borderline, perhaps because of small sample size. The only significant fixed effects were group (*p *< 0.0001) and education (poorer education had lower height by 0. 71 cm than higher levels of education, *p *= 0.0508). In addition there was an effect of age for children, with each year of age adding 5.25 cm in height (*p *< 0.0001).

**Table 9:** Estimates and standard errors of covariates in the best fitting mixed models fitted to height

Effect	Estimate	Standard error	*p*-value		Correlation	95% CI	*P*-value
**Model a) Fitted to maternal line only ‡**							

Education 0&1 vs 2	-0.71	0.363	0.0508	Child and Mother	0.352	(0.271,0.427)	<0.0001

Effect of age within children				Child and MGM	0.164	(0.007, 0.312)	0.0403

Age*child	5.25	0.631	<0.0001	Child and MGF	0.25	(0.051, 0.428)	0.0137

No effect of age in other groups.				Mother and MGF	0.431	(0.243, 0.584)	<0.0001

**Model (b) Paternal line only ‡**							

Effect of age within group.							

age*Child	5.28	0.634	<0.0001	Child and father	0.509	(0.141, 0.744)	0.0071

age*PGM	-0.12	0.075	0.0957	Child and PGM	0.283	(0.078, 0.463)	0.0069

age*PGF	-0.19	0.099	0.0586	Child and PGF	0.264	(0.007, 0.486)	0.0427

No effect of age in other groups.				PGM and PGF	0.376	(0.049, 0.624)	0.0231

Also shown are the significant (*p *< 0.05) correlation estimates between family members.

MGM: Maternal grandmother, MGF: Maternal grandfather, PGM: Paternal grandmother, PGF: Paternal grandfather

*‡ *Model with vs without correlation terms *p *< 0.0001

Note: Sample size too small to establish a correlation between father and PGF or PGM

#### Height paternal line

Fitting the same model to the paternal line only, again the same correlation structure fitted best (Table 9b). There were significant correlations between the child and all family members with overall *p*-value < 0.0001. Applying the Bonferroni correction, the correlations between child and PGF are no longer significant. Due to missing values on covariates, sample sizes were too small to establish a correlation between father and PGF or PGM within the model. The only significant fixed effects were group and age within group. For children each year of age added 5.28 cm in height (consistent with maternal height model (Table 9a)) *p *< 0.0001. For PGM each year of age lowered height by 0.125 cm (*p *= 0.0957 borderline significance) and for PGF each year of age lowered height by 0.19 cm (*p *= 0.0586).

## Discussion

This analysis reports the first evidence of the parental-offspring BMI associations across three generations where mothers were found to have a stronger BMI association with their progeny. We also compared the cross-generation pattern for BMI with the cross generation pattern for height across three generations. While only the maternal line indicates a cross-generation BMI transmission, both maternal and paternal lines appear to contribute to offspring height. These results confirm previous findings [6,9,10] where a considerable amount of the variance of BMI is related to genetic and shared environmental factors but there remains a proportion that is directly attributable to the maternal line of the family. Our results show for the first time that maternal influence is present over three generations. BMI in early childhood is, arguably, therefore influenced to some degree by maternal specific effects, possibly due to intrauterine exposures, which is in keeping with evidence from animal studies [4].

To our knowledge only two previous human studies have reported overweight and obesity in three generations. In the Belgian-Luxembourg study children's BMI was related to BMI of both parents and obesity measurements in their grandparents [15]. However the measure of obesity in grandparents used in the study was an alternative measure to height and weight and may not accurately reflect the strength of the association. Davis et al. in their study of 2591 US children grouped parental data and grandparental data to generate composite measures of BMI [16] which does not distinguish between the maternal and paternal lines. Neither does it allow simultaneous estimation of the effect of explanatory variables as described here.

Several longitudinal studies have examined BMI across two generations. The British 1958 birth cohort demonstrated how parental BMI during childhood and adulthood was clearly associated with offspring BMI [29]. However, the relative contributions of the parents could not be examined as BMI data was only available for the cohort member and not their partner. In addition BMI zscores were used which has inherent disadvantages. In our mixed model analysis, this problem is solved simply by the inclusion of a group term in the models and by modelling different variances in groups via the correlation structure.

Davey Smith and colleagues examined the BMI relationship between parent and child and found no difference in the relative contributions of maternal or paternal BMI to offspring BMI [5]. Indeed, if the heritability estimates of mothers' BMI with both of her parents in the present study were reported alone, this would indicate the paternal and maternal effects contribute equally to offspring BMI. Furthermore, the maternal effects are stronger between the older generation compared with the younger. The differences in findings between other studies [5] and those presented here may be explained by the specific ages of offspring under study. Between five and seven years children experience varying changes in growth, defined by the critical period of adiposity rebound [30]. We speculate that the age of onset of this growth period may be determined by maternal effects with a certain window of influence from birth to adiposity rebound. Therefore our findings of a maternal specific heritability of BMI may be particularly evident in early childhood.

Findings from the Early Bird 43 study have demonstrated a gender specific association between mothers and daughters and fathers and sons from the ages of 5 to 8 years [6]. We did not find evidence of a gender effect on the child but the findings from the Early Bird study indicate the possible behavioural role models of parents which appear to strengthen with age [6,15,31]. The association between mother and offspring may not be evident in the Early Bird 43 study as firstly the mother's BMI was recorded in cross-section when the child was aged 5 and secondly older children will have longer exposure to environmental factors shared with their parents and therefore demonstrate stronger associations with their parents than younger children [15,31].

Several studies examine the relationship of mother-offspring BMI, birth weight and other anthropometric measures [2,32]. Fewer studies include fathers which limits the interpretation of their results; inclusion of fathers provides a greater understanding of the genetic effects as the father-child relationship has less opportunity for confounding with environmental effects. The heritability estimates of height in the present analysis are clearly larger than for BMI indicating a strong genetic contribution to height. Moreover, the use of height in this study enabled us to demonstrate that the dataset is large enough to differentiate the patterns of BMI and height between generations.

Our study supports previous evidence of the social patterning of height but not of BMI which is in contrast to previous studies [33,34] and furthermore markers of diet and exercise were no longer significant. It is possible that these indicators may be too crude to elucidate clear lifestyle differences, however most measures of diet and physical activity will be influenced by socioeconomic status. What is clear from our findings is that despite the inclusion of socioeconomic indicators there remains a fundamental link between the BMI of mother and child that is not explained by shared environmental factors alone. Furthermore, although certain risk factors were not found to be significant in these models the strong family component would suggest that modifiable risk factors, such as physical activity, may be more likely to be successful if they are targeted at the family level rather than at an individual level.

### Strengths and limitations

The advantages of this study are the prospective design with BMI information collected prior to pregnancy and in early childhood, in addition to data from three generations of one family. To our knowledge this is the first prospective study to show intergenerational associations between BMI in the youngest generation and BMI in the previous two generations, with individual measurements for all four grandparents.

Ideally, the strength of our findings could be improved by a higher response rate and larger cohort numbers. Nevertheless, in a previous analysis we did not find any differences in pre-pregnancy BMI of the mothers who responded to the follow-up compared to non-responders. Furthermore, an investigation into the pattern of missing data did not demonstrate any systematic variability. A potential limitation of this study was that self-reported adult height and weight measures were used and, while such measures are acceptable, may lead to underreporting bias particularly in women [35,36]. This means in fact we may be under-estimating the strength of the effects found since the actual BMI of mothers and grandmothers is likely to be higher.

In epidemiological terms this is a relatively small cohort however there are no comparable cohorts with seven members of the one family. Limited families within the study have complete information on all family members but the mixed model maximises the use of the available data. Furthermore, despite the smaller sample numbers of fathers and grandparents the analyses have sufficient power to demonstrate different familial relationships for BMI and height.

## Conclusions

The findings from this study strongly support the contention that early childhood BMI is associated with factors that are unique to the maternal line alone. Familial transmission of height is more typically genetic in pattern. BMI in early childhood may be transmitted through a combination of genetic and intrauterine effects possibly operating via epigenetic mechanisms.

## Abbreviations

AIC: Akaike's Information Criterion; BMI: Body mass index; FFQ: Food frequency questionnaire; GMS: General medical services; MGF: Maternal grandfather; MGM: Maternal grandmother; PGF: Paternal grandfather; PGM: Paternal grandmother; SRH: Self rated health

## Competing interests

The authors declare that they have no competing interests.

## Authors' contributions

CM carried out the data collection for the follow-up study, data analysis, interpretation and drafting of the manuscript. GK performed and interpreted the mixed model statistical analysis. RT participated in the design of the study and data analysis. CK conceived of the study, participated in its design and coordination and the interpretation of the data analysis. All authors read and conducted a critical revision of the manuscript and approved the final manuscript.

## Funding

All sweeps of the Lifeways Cross-Generation Cohort Study and the PhD fellowship were funded by the Health Research Board (HRB) of Ireland. The Lifeways study is is part of a HRB Centre for Health and Diet Research. The collection of anthropometric measurements at follow up was funded by the Population Health Directorate within the National Health Service Executive. The funding agencies had no role in the design and conduct of the study, in the collection, analysis, and interpretation of the data; or in the preparation, review, or approval of the manuscript.

## Pre-publication history

The pre-publication history for this paper can be accessed here:

http://www.biomedcentral.com/1471-2458/12/81/prepub
